# Five Complete Chloroplast Genome Sequences from *Diospyros*: Genome Organization and Comparative Analysis

**DOI:** 10.1371/journal.pone.0159566

**Published:** 2016-07-21

**Authors:** Jianmin Fu, Huimin Liu, Jingjing Hu, Yuqin Liang, Jinjun Liang, Tana Wuyun, Xiaofeng Tan

**Affiliations:** 1 Key Laboratory of Cultivation and Protection for Non-Wood Forest Trees, Ministry of Education, Central South University of Forestry and Technology, Changsha, Hunan, China; 2 Non-Timber Forestry Research and Development Center, Chinese Academy of Forestry, Zhengzhou, Henan, China; 3 Department of Bioinformatics, Haplox Biotechnology Co., Ltd., Shenzhen, China; Austrian Federal Research Centre for Forests BFW, AUSTRIA

## Abstract

*Diospyros* is the largest genus in Ebenaceae, comprising more than 500 species with remarkable economic value, especially *Diospyros kaki* Thunb., which has traditionally been an important food resource in China, Korea, and Japan. Complete chloroplast (cp) genomes from *D. kaki*, *D. lotus* L., *D. oleifera* Cheng., *D. glaucifolia* Metc., and *Diospyros* ‘Jinzaoshi’ were sequenced using Illumina sequencing technology. This is the first cp genome reported in Ebenaceae. The cp genome sequences of *Diospyros* ranged from 157,300 to 157,784 bp in length, presenting a typical quadripartite structure with two inverted repeats each separated by one large and one small single-copy region. For each cp genome, 134 genes were annotated, including 80 protein-coding, 31 tRNA, and 4 rRNA unique genes. In all, 179 repeats and 283 single sequence repeats were identified. Four hypervariable regions, namely, intergenic region of *trnQ*_*rps16*, *trnV*_*ndhC*, and *psbD*_*trnT*, and intron of *ndhA*, were identified in the *Diospyros* genomes. Phylogenetic analyses based on the whole cp genome, protein-coding, and intergenic and intron sequences indicated that *D. oleifera* is closely related to *D. kaki* and could be used as a model plant for future research on *D. kaki*; to our knowledge, this is proposed for the first time. Further, these analyses together with two large deletions (301 and 140 bp) in the cp genome of *D*. ‘Jinzaoshi’, support its placement as a new species in *Diospyros*. Both maximum parsimony and likelihood analyses for 19 taxa indicated the basal position of Ericales in asterids and suggested that Ebenaceae is monophyletic in Ericales.

## Introduction

*Diospyros*, belonging to Ebenaceae, is a large genus with more than 500 species that are distributed worldwide [1]. *D. kaki* is the most important economic crop and the most widely cultivated species of *Diospyros*. It is believed to have originated in China and has been an important food source in China, Korea, and Japan from prehistoric times [2]. The fruit of *D. kaki* is delicious and has an extensive popularity globally. In 2013, the global production of persimmon (*D. kaki*) was 4,637,357 tons, of which 78.0% was from China [3]. In addition, the fruit is used as a source of persimmon lacquer and tannin [4]. The leaves can be used as tea and are known to have phytochemical and pharmacological properties [5, 6]. At present, about 1000 cultivars exist in China [7], most of which are hexaploid, while some are nonaploid [8]. The progenitor, origin, and polyploidization mechanisms of *D. kaki* are still ambiguous; thus, identifying a closely related diploid species to be used as reference for future research is necessary. Previous studies indicated that the diploid species—*D. oleifera*, *D. lotus*, *D. glaucifolia*, and *D*. ‘Jinzaoshi’—are the related species of *D. kaki* [9, 10]. They are also widely used species of *Diospyros*. *D. glaucifolia* is used as timber wood; *D. oleifera* is used as a source of tannin, whereas *D. lotus* and *D*. ‘Jinzaoshi’ are cultivated for their fruits. *D*. ‘Jinzaoshi’, known as Jinzaoshi in China, is a controversial species. It has been accepted as a cultivar of *D. kaki*, but recent studies based on morphological as well as internal transcribed sequence (ITS) and *matK* sequence analyses proposed that *D*. ‘Jinzaoshi’ might be a new species [11].

In addition to these factors, the classification of *Diospyros* is very difficult because of the natural or artificial interspecific hybrids, indistinguishable morphological features across species, and the complex chromosome numbers (2n = 2X, 4X, 6X, 9X = 30, 60, 90, 135) [8]. The identification of the phylogenetic relationship of *Diospyros* has been attempted using various methods based on morphological characteristics [12] and molecular markers [13, 14]. Different markers yield inconsistent results, probably because of the discrepant sequence divergence ratios and tree-generating methods used. Additional markers should be detected to reveal the accurate relationship within *Diospyros* and to elucidate phylogeny within the asterids.

The chloroplast (cp) genome of higher plants has a conserved quadripartite structure with one large single-copy region (LSC: 80–90 kb) and one small single-copy region (SSC: 16–27 kb) separated by two identical inverted repeat regions (IR: 20–28 kb in length) [15, 16]. The gene content and gene order in angiosperm cp genomes are usually highly conserved, containing 110–130 distinct genes that encode 4 rRNAs, 30 tRNAs, and 80 protein-coding genes [17]. However, the angiosperm cp genome has also undergone several large mutations such as genome rearrangement and gene loss and gain in both monocots [18] and dicots [19].

Cp genomes are useful in taxonomy and evolutionary studies [20, 21] for their small size, conserved gene content and arrangement, and maternally inherited characteristics [22, 23]. The basal asterids Ericales are a large order containing more than 20 families [24]. However, complete cp genomes have been sequenced from only four families (Ericaceae, Theaceae, Actinidiaceae, and Primulaceae) [25–28]. Analysis of more cp genomes is needed for an accurate phylogeny of angiosperms. The cp genome can also be used in genetic transformation [29], agricultural trait improvement [30], and DNA barcoding [31]. Cp genome transformation is superior to nuclear transformation because of its high level of transgene expression and gene containment [32]. Complete cp genome of *Diospyros* or Ebenaceae has not yet been sequenced despite their remarkable economic value.

In this study, we sequenced complete cp genomes from five species of *Diospyros* and conducted comparative analyses within both *Diospyros* and Ericales. The comparative analyses of the cp genomes of Ebenaceae and four other families with published cp genomes were conducted to elucidate the phylogeny and genomic structures of Ericales.

## Materials and Methods

### Plant Materials

Healthy and young leaves were collected from adult plants of five species, *D. kaki*, *D*. ‘Jinzaoshi’, *D. glaucifolia*, *D. lotus*, and *D. oleifera*, grown in a field nursery in Yuanyang County, China. This nursery is a germplasm collection center of *Diospyros* species owned by Non-timber Forestry Research and Development Center, Chinese Academy of Forestry. Our study was permitted and approved by this authority. No endangered or protected species were sampled.

### DNA Sequencing, Genome Assembly, and Validation

Total DNA was extracted from 50 g of fresh leaves using a DNeasy Plant Mini Kit (Qiagen, Valencia, CA, USA). After purification, the DNA sample was randomly fragmented to construct paired-end (PE) libraries according to the Illumina preparation manual (San Diego, CA, USA). This sequencing technology was chosen because of its high accuracy in homopolymer sequencing [33] and its wide application to other plastomes [34, 35]. Accurate sequencing of mononucleotide repeats is important since they have variable lengths in different haplotypes [36].

The cp DNA was assembled as follows: all reads were filtered by trimming 20 bp from the PE reads and reads with quality score of less than 20. The clean PE reads were overlapped using FLASH ver. 1.2.6 [37] and then aligned to the cp database by using Burrows–Wheeler Aligner (BWA) software [38]. Celera Assembler [39] was used to assemble the reads into contigs, which were then scaffolded using SSPACE [40]. Mapping assembly was generated using LASTZ [41] and *Camellia yunnanensis* (NC_013707) as a reference sequence.

The gaps were filled using GapFiller [42] to obtain the complete genomes. The complete cp genome sequences were validated by designing 101 pairs of primers to obtain PCR products. Five of these primers covered the four junctions between single-copy (SC) and inverted-repeat (IR) regions. The PCR products were sequenced using Sanger sequencing and aligned to *Diospyros* cp genomes. These complete cp genomes were deposited in GenBank (S1 Table).

### Gene Annotation and Repeat Identification

Gene annotation was conducted using the Dual Organellar GenoMe Annotator (DOGMA) [43]. The final annotation was obtained by manual correction based on published cp gene annotations deposited in online databases. The circular gene distribution map was drawn using OGDraw [44].

Four types of repeats—forward, reverse, complement, and palindromic—were assessed using REPuter [45] with the minimal repeat size of approximately ≥ 20 bp. Microsatellites were detected using MISA; they were defined as (unit size/minimum number of repeats) 1/10, 2/6, 3/5, 4/4, 5/3, and 6/3 [46].

### Phylogenomic Analyses

Unless otherwise specified, all the multiple sequence alignments in this study were performed using Clustalw v2.0.12 with default parameters. The maximum parsimony (MP) trees were reconstructed using PAUP* v4.0b10 [47] with heuristic search and tree-bisection-reconnection (TBR) for branch-swapping settings. Gaps and multistate taxa were treated as missing and uncertainty, respectively. One tree was held at each step during stepwise addition. The MulTrees option was set in effect, and Steepest descent was not in effect. Before maximum likelihood (ML) analyses, the target alignment was uploaded to Cipres to identify the best model by using the Akaike information criterion (AIC) implemented in the jModelTest2 program [48]. The ML trees were reconstructed with RAxML v8.2.6 using the corresponding best model [49]. In both the MP and ML trees, bootstrap analyses were performed with 1000 replicates [50].

## Results

### Genome Sequencing, Assembly, and Validation

Overall, 477–1,150 million bp short reads were produced by sequencing of the five species on the Illumina Hiseq and Miseq platform. The short reads were aligned against the reference cp genome, and a total of 18.2–58.9 million bp were mapped to the reference genome, with an average of 116–376× read depth (S1 Table).

A total of 101 pairs of primers were designed to validate the genome assemblies, including the junctions between four regions in *Diospyros* cp genome (S2 Table). After PCR and Sanger sequencing, the sequences were aligned directly against the *Diospyros* genomes to correct for nucleotide mismatches or indels.

### Genome Features

**Fig 1:**
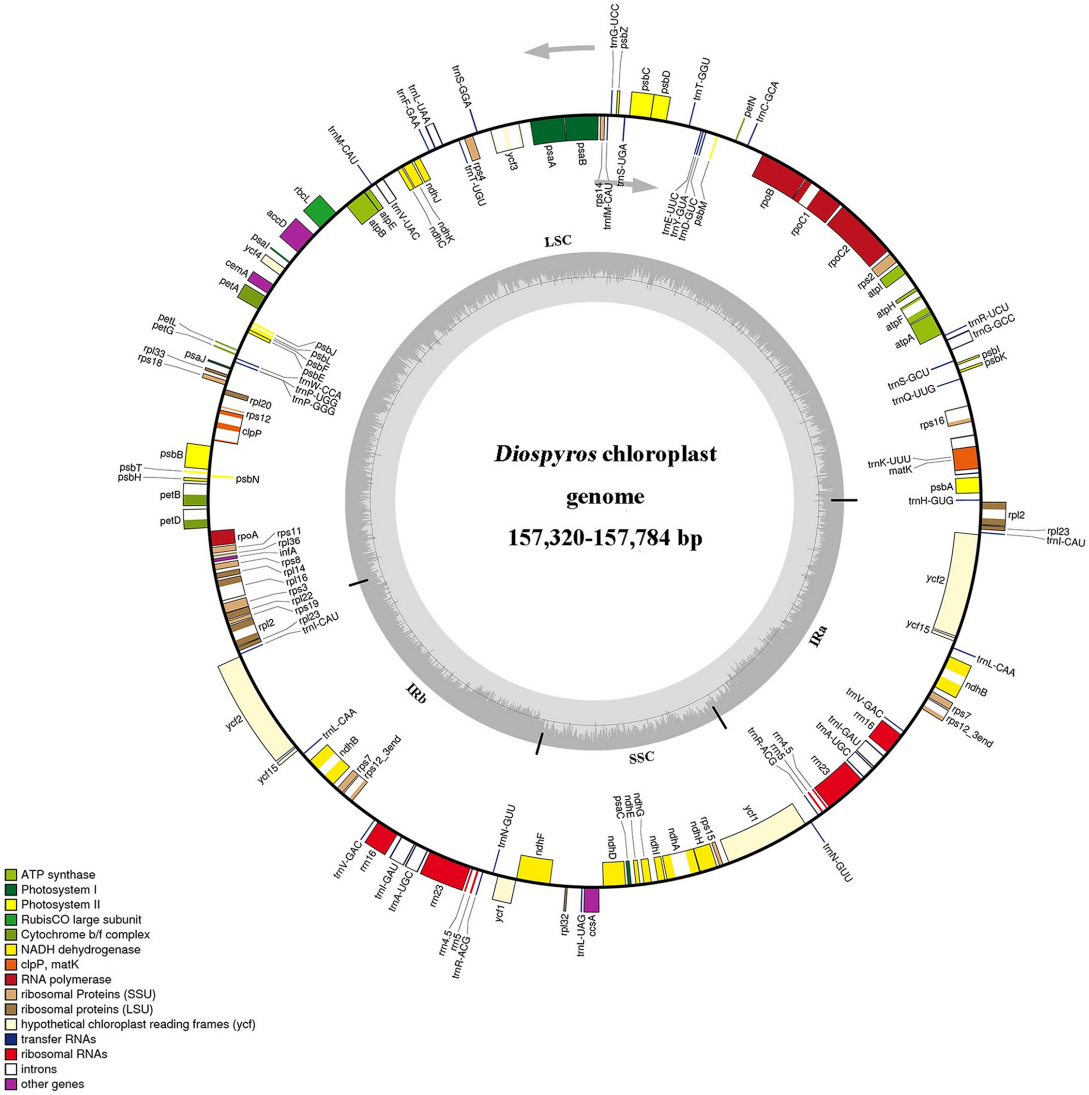
Gene maps of *Diospyros* chloroplast genomes. Genes on the outside of the large circle are transcribed clockwise and those on the inside are transcribed counterclockwise. The genes are color-coded based on their function. Dashed area represents the GC composition of the chloroplast genome.

*Diospyros* cp genomes consist of two IRs (26,079–26,119 bp) segregated by two SC regions, namely, LSC (86,948–87,059 bp) and SSC (18,076–18,532 bp), thereby presenting a typical quadripartite structure (Fig 1, S3 Table). The genome structure and gene content and order were identical in the five *Diospyros* cp genomes. For each of the five *Diospyros* cp genomes, 134 functional genes were predicted (Table 1), of which 115 were unique genes (including 80 protein-coding genes, 31 transfer RNA genes, and 4 ribosomal RNA genes), and 19 were duplicated genes in the IR regions. Eighteen distinct genes contained one intron, two of which contained two introns. The *rps12* gene, similar to *Actinidia chinensis* [27], is a trans-spliced gene with the 5′ end located in the LSC region and the duplicated 3′ end in the IR region. As has been reported previously in other plants [51–53], we also detected several non-canonical start codons, e.g., ACG and GTG, in *ndhD* and *rps19*, respectively.

**Table 1:** Genes located on *Diospyros* chloroplast genomes.

Category	Gene name
Ribosomal RNAs	*rrn16*(2), *23*(2), *4.5*(2), *5*(2)
Transfer RNAs	*trnA-UGC(2), *C-GCA*, *D-GUC*, *E-UUC*, *F-GAA*, *G-GCC, *G-UCC*, *H-GUG*, *I-CAU*(2), *I-GAU(2), *K-UUU, *L-CAA*(2), *L-UAA, *L-UAG*, *M-CAU*, *N-GUU*(2), *P-UGG*, *P-GGG*, *Q-UUG*, *R-UCU*, *R-ACG*(2), *S-UGA*, *S-GCU*, *S-GGA*, *T-GGU*, *T-UGU*, *V-GAC*(2), *V-UAC, *W-CCA*, *Y-GUA*, *fM-CAU*
Proteins of the small ribosomal subunit	*rps2*, *3*, *4*, *7*(2), *8*, *11*, *12(2), *14*, *15*, *16, *18*, *19*
Proteins of the large ribosomal subunit	*rpl2(2), *14*, *16, *20*, *22*, *23*(2), *32*, *33*, *36*
Subunits of RNA polymerase	*rpoA*, *B*, *C1, *C2*
Subunits of NADH- dehydrogenase	*ndhA, *B(2), *C*, *D*, *E*, *F*, *G*, *H*, *I*, *J*, *K*
Subunits of Photosystem I	*psaA*, *B*, *C*, *I*, *J*
Subunits of Photosystem II	*psbA*, *B*, *C*, *D*, *E*, *F*, *H*, *I*, *J*, *K*, *L*, *M*, *N*, *T*, *Z*
Large subunit of Rubisco	*rbcL*
Subunits of cytochrome b/f complex	*petA*, *B, *D, *G*, *L*, *N*
Subunits of ATP synthase	*atpA*, *B*, *E*, *F, *H*, *I*
Acetyl-CoA carboxylase	*accD*
Cytochrome c biogenesis	*ccsA*
Maturase	*matK*
Protease	**clpP
Envelope membrane protein	*cemA*
Conserved hypothetical chloroplast reading frames	*ycf1*(2), *2*(2), **3, *4*, *15*(2)
Translation initiation factor	*infA*

(2) indicates genes that have undergone duplication. * and ** indicate genes containing one and two introns, respectively.

The expansion of *ycf1* into the IRa region is attributed to the formation of the *ycf1* pseudogene at the corresponding border of IRb and SSC (Fig 1). Such expansion has been detected in other angiosperm plastid genomes [51].

In total, 58% of the *Diospyros* cp genomes represented coding regions, whereas the remaining 42% were non-coding regions.

### Repetitive Sequence

Four repeat types—forward, reverse, palindromic, and complement—were detected using REPuter [45]. The length and similarity of these sequences were more than 20 bp and 90%, respectively (S4 Table). We identified 179 repeats in the five *Diospyros* cp genomes, 100 of which were shared by all the genomes, and four, five, seven, and two repeats were specifically detected in *D. kaki*, *D. oleifera*, *D*. ‘Jinzaoshi’, and *D. glaucifolia*, respectively. Palindromic repeats were the most common, accounting for 49%, followed by forward repeats (40%) and reverse repeats (10%). Only one complement repeat (20 bp) was specifically identified in the LSC region in the *D*. ‘Jinzaoshi’ genome. Except for a few repeats in the coding regions of *ycf2*, *ndhH*, *ndhC*, *trnS-GCU*, *trnS-UGA*, *trnfM-CAU*, *trnV-UAC*, *trnS-GGA*, *trnP-GGG*, and *trnA-UGC*, the majority were located in the noncoding regions.

In total, 53, 52, 61, 55, and 62 single sequence repeat (SSR) loci were identified in *D. kaki*, *D*. ‘Jinzaoshi’, *D. lotus*, *D. oleifera*, and *D. glaucifolia* cp genomes, respectively (S5 Table). Among all mononucleotide repeats, 278 were A/T stretches, whereas only one C stretch was found in the *D. locus* and one G stretch was found in the *D. glaucifolia* cp genome. Three tetranucleotide repeats (AAAT) were found only in the *D. kaki*, *D*. ‘Jinzaoshi’, and *D. oleifera* cp genomes. Di-, tri-, penta-, or hexanucleotide repeats were not found. Most of the SSRs were located in the LSC (209) region, followed by those in the SSC (49) and IR (25) regions, and 67% were intergenic sequences.

### Comparison of the Whole Chloroplast Genomes among Ericales

**Fig 2:**
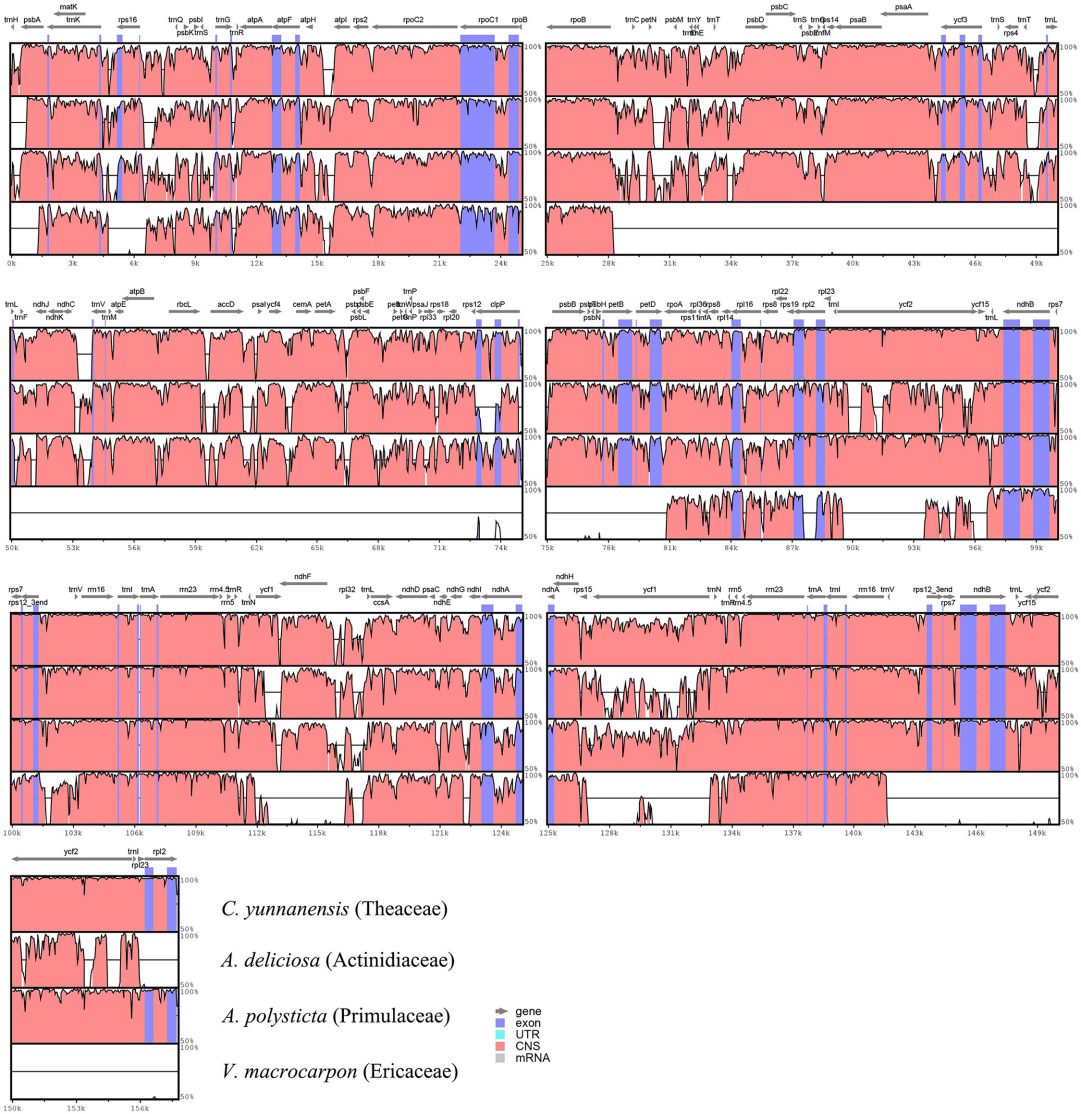
Global alignment of Ebenaceae genome and other published chloroplast genomes in Ericales using VISTA. Y-axis indicates the range of identity (50%–100%). Alignment was performed using *D. kaki* as a reference.

The global alignments between Ebenaceae and other published families in Ericales were performed using mVISTA [54] (Fig 2). The cp genome of *Vaccinium macrocarpon* in Ericaceae was remarkably different from that of Ebenaceae. IRs were more conserved than SCs. Unlike coding sequences, non-coding sequences exhibit a higher divergence across different species. The intergenic regions of *trnQ*_*rps16*, *atpI*_*atpH*, *psbJ*_*petA*, *ndhF*_*rpl32*, *rpl32*_*trnL*, *trnV*_*ndhC*, and *psbD*_*trnT* were highly variable.

### Indel Identification and Relationship of the Five *Diospyros* cp Genomes

**Fig 3:**
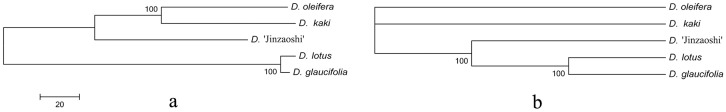
Phylogenetic trees based on whole genome sequences of *Diospyros*. (a) Maximum likelihood tree, (b) Maximum parsimony tree.

All the ML trees reconstructed based on the whole cp genome sequences, protein-coding sequences, and intergenic and intron sequences of *Diospyros* indicated that *D. kaki* was closer to *D. oleifera*, whereas *D. lotus* had a closer relationship with *D. glaucifolia* (Fig 3a, S1a and S2a Figs). MP trees reconstructed using corresponding sequences were consistent with the ML tree topology (Fig 3b, S1b and S2b Figs).

**Fig 4:**
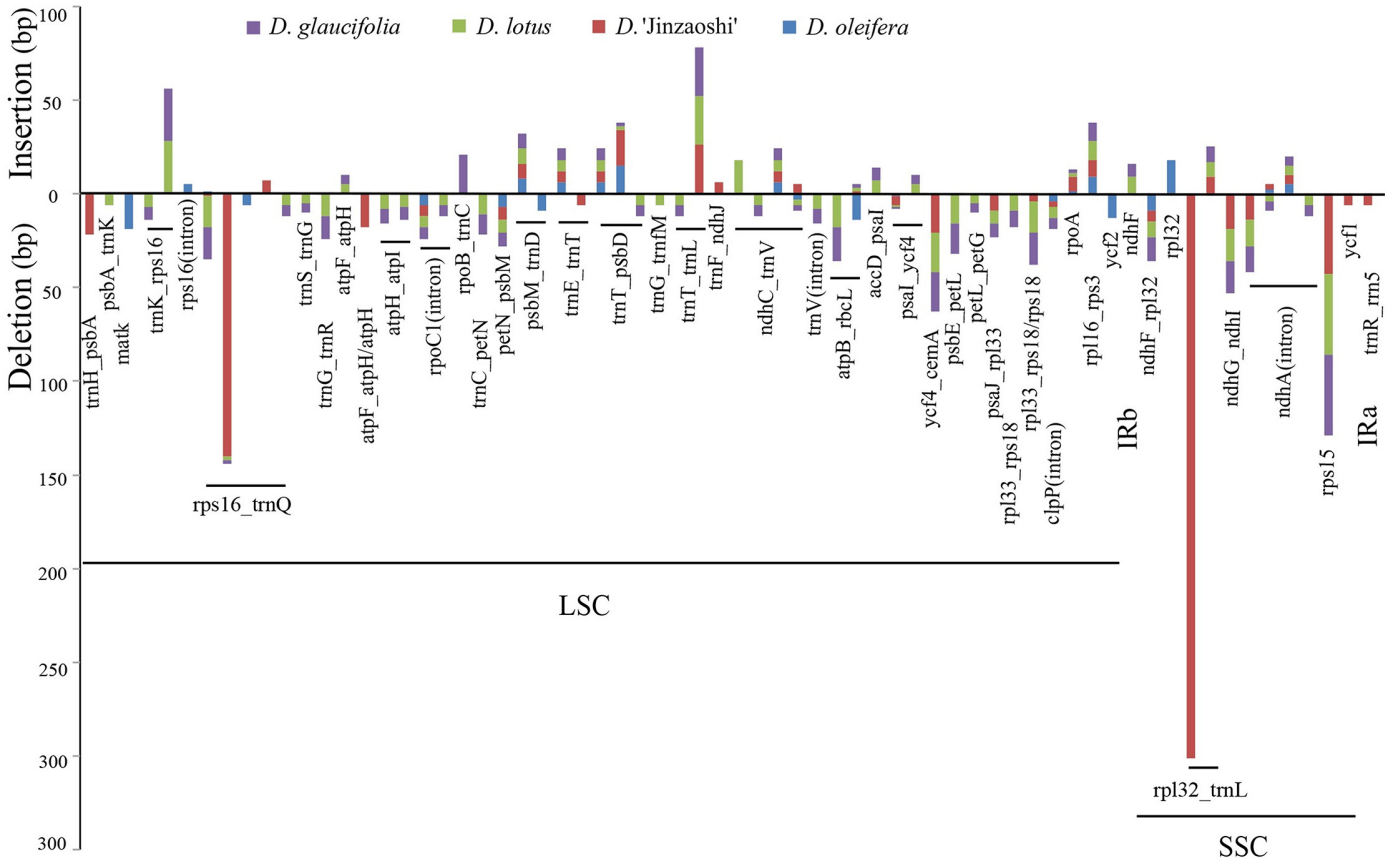
Indels (≥5 bp) identified based on multiple sequence alignment of five *Diospyros* cp genomes. Insertions are shown above and deletions below the horizontal axis. Indel distribution was positioned using *D. kaki* as a reference.

Multiple sequence alignment was performed, and indels more than 5 bp long were detected to reveal the variations within the five *Diospyros* cp genomes (Fig 4). Although the five *Diospyros* cp genomes were highly conserved, the existing differences might reveal species variation and differentiation. In total, 66 loci were identified, and the intergenic region *trnQ*_*rps16* with five loci was the most variable region, followed by *trnV*_*ndhC*(4), *ndhA*intron (4), and *psbD*_*trnT*(3). The two largest indels were the deletions of 140 bp and 301 bp located in *trnQ*_*rps16* and *rpl32*_*trnL* in the cp genome of *D*. ‘Jinzaoshi’, respectively. Both MP and ML trees based on the sequences of these four hypervariable regions corroborated the results based on whole cp genome sequences (S3a and S3b Fig).

### Analysis of IRs

**Fig 5:**
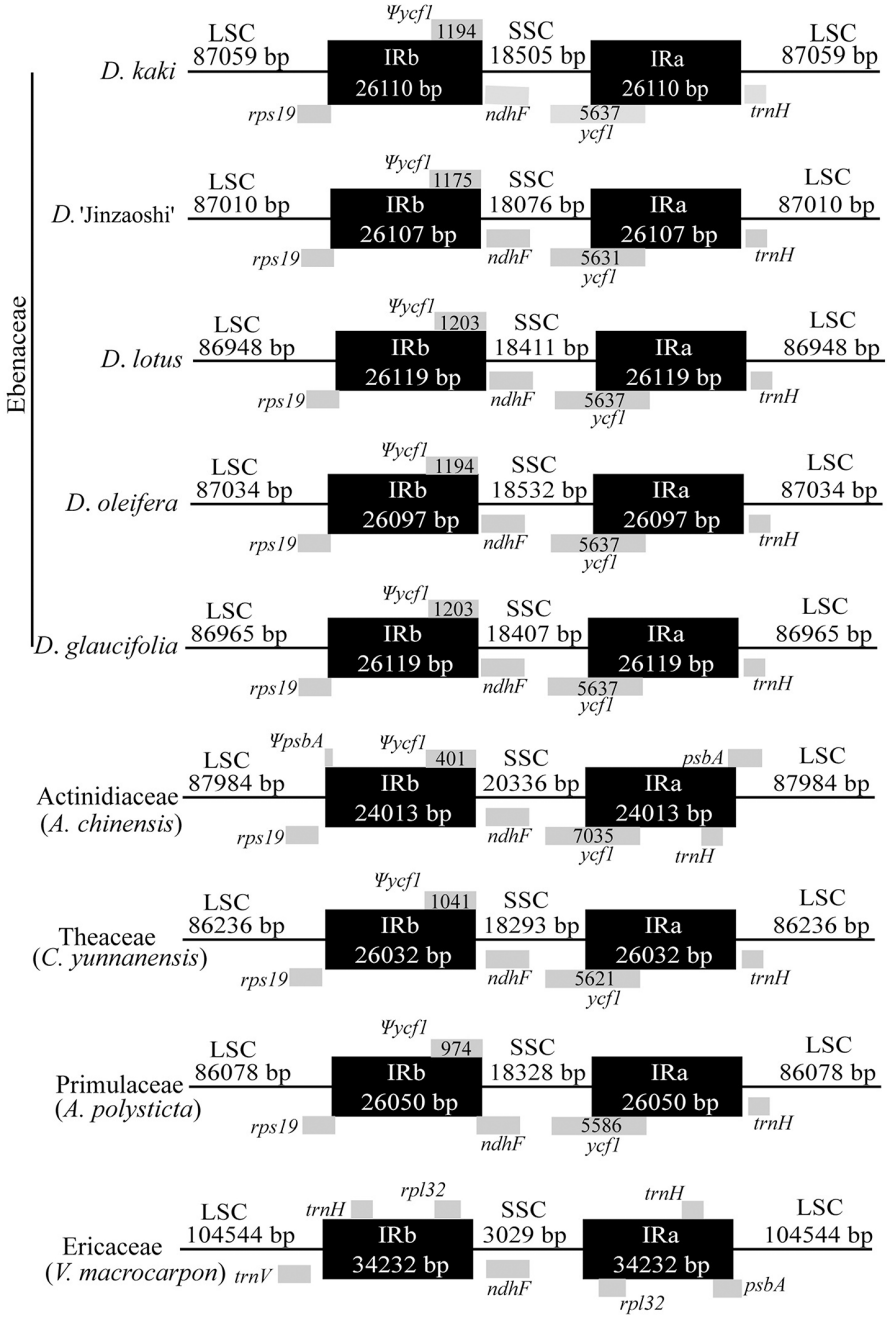
The comparison of inverted-repeat (IR) and single-copy (SC) borders among nine chloroplast genomes. Gene annotation or portions are represented by gray boxes above or below.

In Ebenaceae, the IRa/SSC borders were located in the 3′ region of the *ycf1* gene creating the *ycf1* pseudogene at the IRb/SSC border (Fig 5). This finding is similar with those in Actinidiaceae, Theaceae, and Primulaceae but remarkably different from that in Ericaceae. The IRb/SSC borders were located upstream of the *ndhF* gene, except in Primulaceae whose IRb/SSC junction was located in the 5′ region of *ndhF*. In Ebenaceae, the IRa/LSC junctions were located in the upstream region of *trnH*-GUG, similar to that in Theaceae. However, this gene was found in the IRs in Actinidiaceae and Ericaceae, as well as in most monocot cp genomes [55]. In Ebenaceae and Primulaceae, the IRb/LSC junctions were located within *rps19*, but no copy was generated in the corresponding region.

### Phylogenetic Analysis

**Fig 6:**
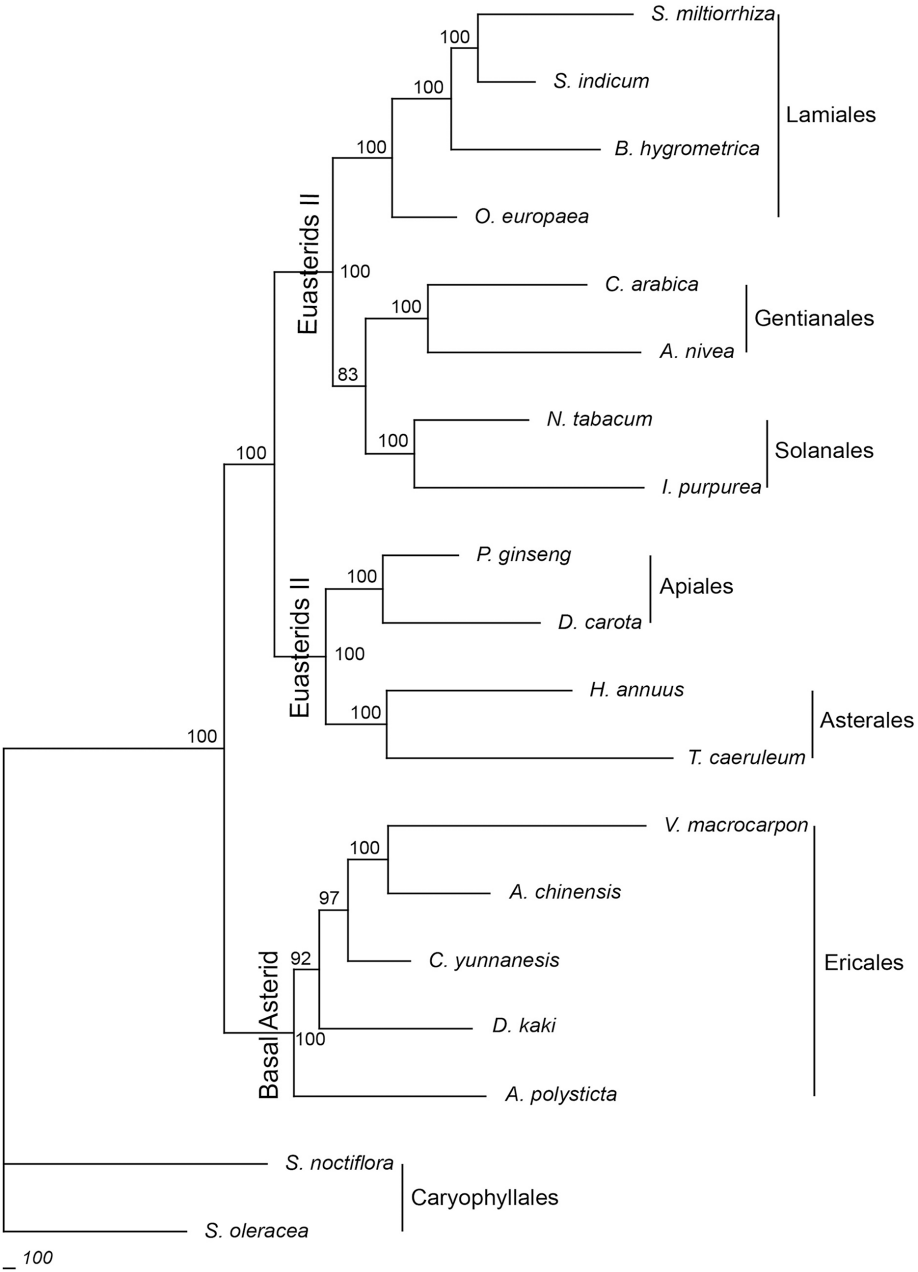
Phylogenetic tree of the asterid clade. The tree was reconstructed based on 61 protein-coding sequences shared by 19 angiosperm species. The numbers at the nodes indicate bootstrap values (1000 replications).

The phylogenetic relationship between *Diospyros* and other asterids was determined by collecting 18 published cp genome sequences from the GenBank of the NCBI database (S6 Table). Two cp genome sequences from *Spinacia* and *Silene* belonging to Caryophyllales were included as outgroup taxa. Sixty-one protein-coding sequences shared by these cp genomes were aligned in a single data matrix with a total of 52,294 characters included. Of all the characters, 35,097, 8414, and 8783 were constant, variable, and parsimony-informative, respectively. All the nodes in the phylogenetic tree received high bootstrap (83%–100%). The MP tree strongly indicated that Ericales is a basal sister order to the subdivision of euasterids (euasterids I and II; Fig 6) and suggested the monophyletic placement of Ebenaceae in Ericales. Lamiales, Solanales, and Gentianales were clustered into the subdivision of euasterids I, whereas Apiales and Asterales were included in euasterids II. The tree topology reconstructed using the ML method was consistent with the MP tree topology (S4 Fig).

## Discussion

In this study, five sequences of *Diospyros* cp genomes were sequenced and validated using PCR-based Sanger sequencing. The complete cp genomes ranged from 157,300 to 157,784 bp, which is within the range of the cp genomes of other angiosperms [51]. Despite the occurrence of frequent large-scale genome rearrangements and gene loss-and-gain events in several lineages of land plants [56, 57], the cp genomes of *Diospyros* were highly conserved with identical gene content and gene order and genome structure comprising four parts, as noted in other angiosperms [58]. Similar to previously published asterid plastid genomes [59, 60], the *Diospyros* cp genome contained more AT and had a GC content of 37%.

SSRs are widely used markers in population genetics [61, 62] and in phylogenic investigations [63, 64] because of their high polymorphism even within species. A total of 283 SSR loci were identified in the five *Diospyros* cp genomes; most of them were intergenic sequences, indicating numerous variations in these regions. Most of the mononucleotide repeats were A/T stretches, contributing to the rich A/T content in the cp genomes of *Diospyros* and suggesting that most of the cp SSRs are short polyadenine (polyA) or polythymine (polyT) repeats [34]. Thus, *Diospyros* cp microsatellites might be useful tools in ecological and evolutionary studies, which warrants further research.

Global alignment between Ebenaceae and other published cp genomes in Ericales indicated that the IR regions were more conserved, probably because of copy correction by gene conversion when mutations are introduced into IRs [65]. The significant difference between the cp genome of *V. macrocarpon* and that of other species might have been caused by multiple structural rearrangements in its cp genome [25]. Seven intergenic regions with rich variation were included in the 13 hotspots reported in the plastid genomes of several plants, including asterids [66]. These regions could be developed as interspecific DNA markers for the phylogenetic analysis in Ericales.

The IR regions play an important role in stabilizing plastid genome structure [67]. Although IRs are highly conserved, IR contraction and expansion events are common in the evolutionary history and are mainly responsible for length mutations of plastid genomes [51, 68]. In this study, we compared the IR/SC junctions within Ericales. The IR/SC junctions of *Diospyros* were similar and showed little difference with those of Actinidiaceae, Theaceae, and Primulaceae. The cp genome of Ericaceae was significantly different from those of others, further confirming the rearrangements during its evolution [25]. Our results indicated that the cp genomes might be conserved in closely related species, whereas species belonging to different families might have greater diversity, such as the large inversions in the cp genome of *Eucommia ulmoides* [69] and one inverted repeat loss in *Astragalus membranaceus* [70].

Phylogenetic trees reconstructed using different sequences indicated the closer relationship between *D. kaki* and *D. oleifera*. This finding is consistent with that of our previous study based on SSR and ITS regions (S5 and S6 Figs) [71, 72] and with that of a study investigating taxonomy based on morphology [73]. The morphological characteristics of *D. oleifera* are similar to those of *D. kaki*: both have pistillate flowers, styles that are parted, and branches without pellicle. However, the branches of *D. lotus* and *D. glaucifolia* are covered with pellicle and pistillate flower styles are parted halfway. In *D*. ‘Jinzaoshi’, the branches are covered with pellicle, but the pistillate flower style is joined (for more details, see [11, 73]). Multiple sequence alignment among five *Diospyros* genomes indicated that most of the indels were intergenic sequences located in the LSC and SSC regions, which is consistent with the findings of previous studies suggesting that SC regions are less conserved than IR regions [58, 74, 75]. The large deletions identified in the cp genome of *D*. ‘Jinzaoshi’ might have been caused by slipped-strand mispairing [76] or illegitimate recombination events [77–79]. The indels identified in the *Diospyros* cp genomes might have numerous important applications in systematics and evolutionary biology, such as elucidating the origin of domesticated species [80], tracing biogeographic movements [81–83], and clarifying complex relationships among species [84]. Furthermore, these hotspot regions could be used to determine the molecular phylogeny of other *Diospyros* species. Previous studies based on morphological as well as ITS and *matK* sequence analyses proposed that “Jinzaoshi” does not belong to *D. kaki* and other related *Diospyros* species and might be a new species of *Diospyros* [11]. The two large deletions in the cp genome of *D*. ‘Jinzaoshi’ and the phylogenetic trees inferred from the five *Diospyros* cp genomes indicated that *D*. ‘Jinzaoshi’ is a new species and should be named in the future.

Both tree topologies reconstructed using the MP and ML methods confirmed the basal position of Ericales in asterids and the subdivision of this clade. This is consistent with the findings of a previous phylogenetic analysis based on the complete cp genomes of 15 asterid species and one outgroup [27]. Thirteen out of 16 nodes in the MP tree received a bootstrap support of 100%, suggesting that proper settings were used during the reconstruction. Ebenaceae was resolved monophyletic, which corroborated the findings of a previous study based on five genes from the plastid and mitochondrial genomes [85]. Numerous studies use DNA sequences from complete cp genomes to estimate phylogenetic classification of angiosperms [86, 87]. Completely sequenced cp genomes comprise abundant phylogenetic information, and several complete cp genome sequences have been successfully applied to study the phylogenetic relationships among angiosperms [21, 87]. Better understanding of the evolutionary history of asterids requires expanded range of sampling.

## Conclusion

To our knowledge, this is the first report of the complete cp genome sequence of Ebenaceae. The sequences of the complete cp genomes of *Diospyros* and sequencing and assembly strategies can be used as a reference for future cp genome sequencing within Ebenaceae, or even Ericales. The available plastid genomes contain sufficient phylogenetic information to resolve interspecific relationships, conduct phylogenetic and classification analyses, and trace the origin of *Diospyros*, in particular, of economically important plants. Since the majority of *D. kaki* are hexaploid, with a few being nonaploid [8], further investigation of its genetic background is challenging, especially the whole-genome sequencing. *D. oleifera* could be considered as a model plant to study *D. kaki* and its cultivars. Furthermore, our study findings confirmed that *D*. ‘Jinzaoshi’ is a new species and indicated that the complete cp sequences might provide a practical and efficient approach to clarify the phylogenetic relationships among *Diospyros* species.

## Supporting Information

S1 Table Statistical analysis of the sequencing information. (XLSX)

S2 Table Primers used for assembly and junction verification. (XLSX)

S3 Table Genomic features of the five *Diospyros* species. (XLSX)

S4 Table Results of the repeated statistical analysis. (XLSX)

S5 Table Single sequence repeats identified in *Diospyros* genomes. (XLSX)

S6 Table Accession numbers of the chloroplast genome sequences used in this study. (XLSX)

S1 Fig Phylogenetic trees reconstructed based on 80 protein-coding sequences of *Diospyros*. (a) Maximum likelihood tree (b) Maximum parsimony tree.
(TIF)

S2 Fig Phylogenetic trees reconstructed based on intergenic and intron sequences of *Diospyros*. (a) Maximum likelihood tree (b) Maximum parsimony tree.
(TIF)

S3 Fig Phylogenetic trees reconstructed based on 4 hypervariable sequences of *Diospyros*. (a) Maximum likelihood tree (b) Maximum parsimony tree.
(TIF)

S4 Fig Maximum likelihood tree reconstructed based on 61 protein-coding sequences shared by 19 angiosperm species. (TIF)

S5 Fig Phylogenetic tree constructed based on the single sequence repeat sequences. (TIF)

S6 Fig Phylogenetic tree constructed based on the internal transcribed spacer region sequences. (TIF)
