# Supplementary figures and images for: Five Complete Chloroplast Genome Sequences from Diospyros: Genome Organization and Comparative Analysis
Source: PLoS One. 2016 Jul 21;11(7):e0159566. doi: 10.1371/journal.pone.0159566 (PMC4956199; doi:10.1371/journal.pone.0159566)

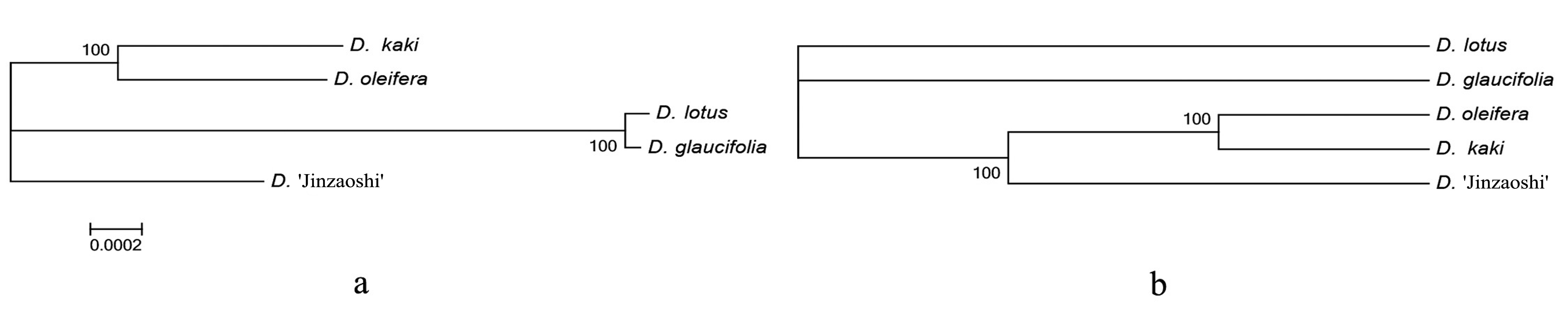

Supplement: S1 Fig — (a) Maximum likelihood tree (b) Maximum parsimony tree. (TIF) [file pone.0159566.s007.tif]

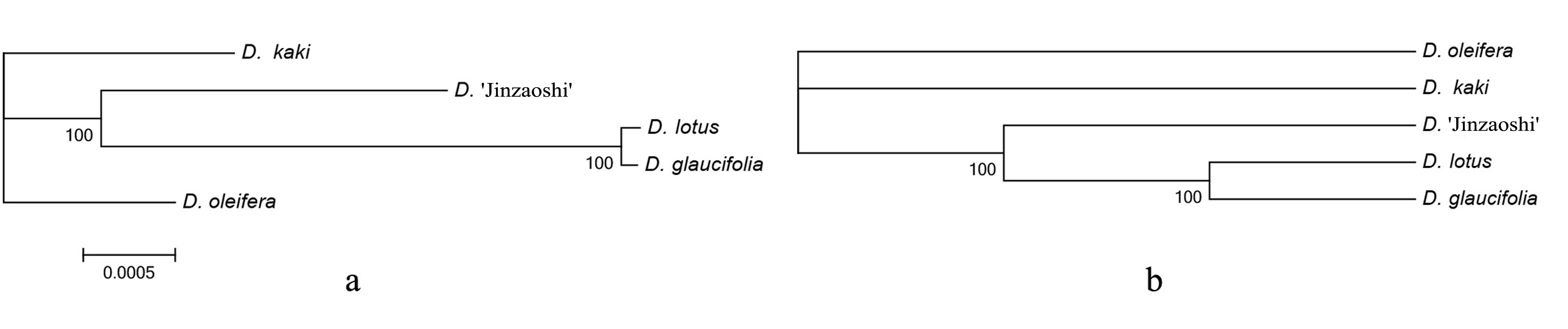

Supplement: S2 Fig — (a) Maximum likelihood tree (b) Maximum parsimony tree. (TIF) [file pone.0159566.s008.tif]

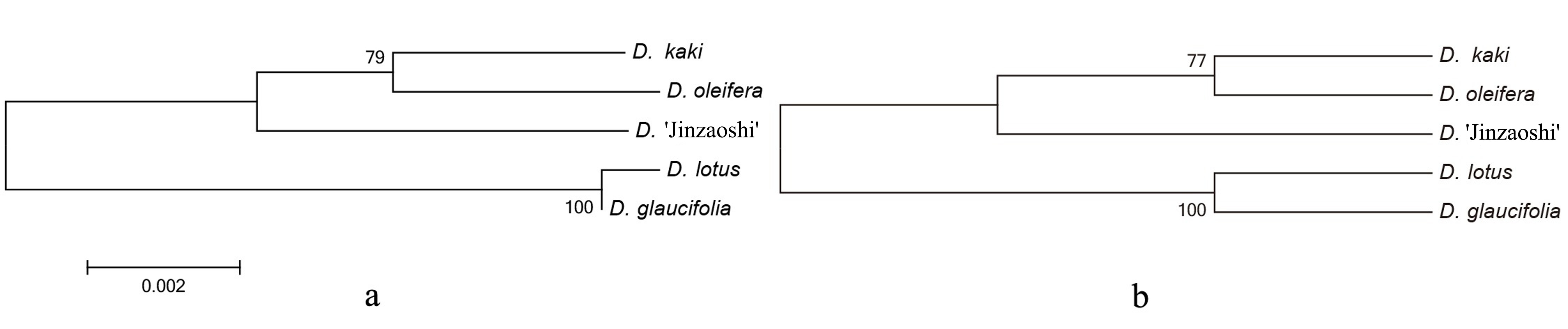

Supplement: S3 Fig — (a) Maximum likelihood tree (b) Maximum parsimony tree. (TIF) [file pone.0159566.s009.tif]

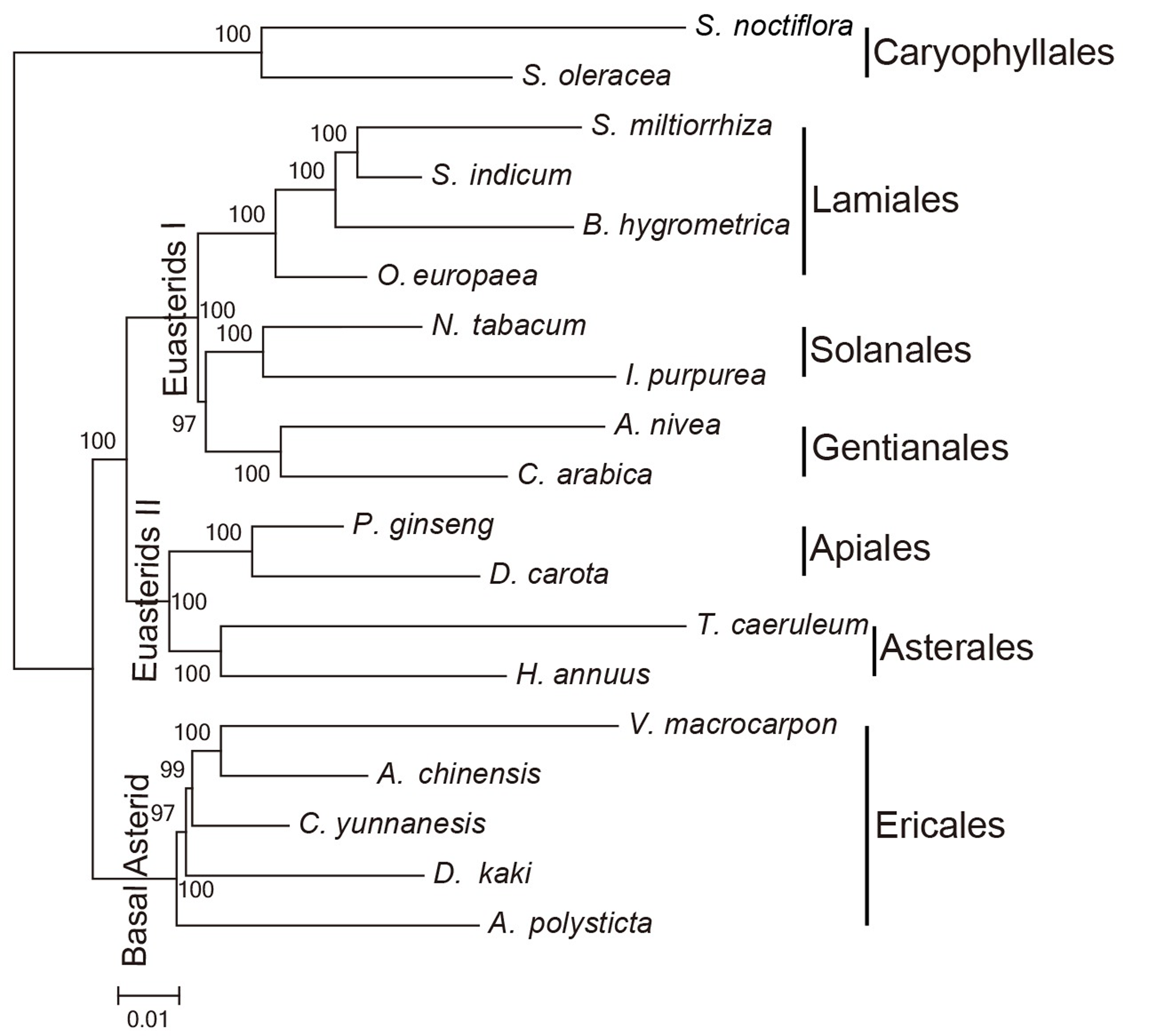

Supplement: S4 Fig — (TIF) [file pone.0159566.s010.tif]

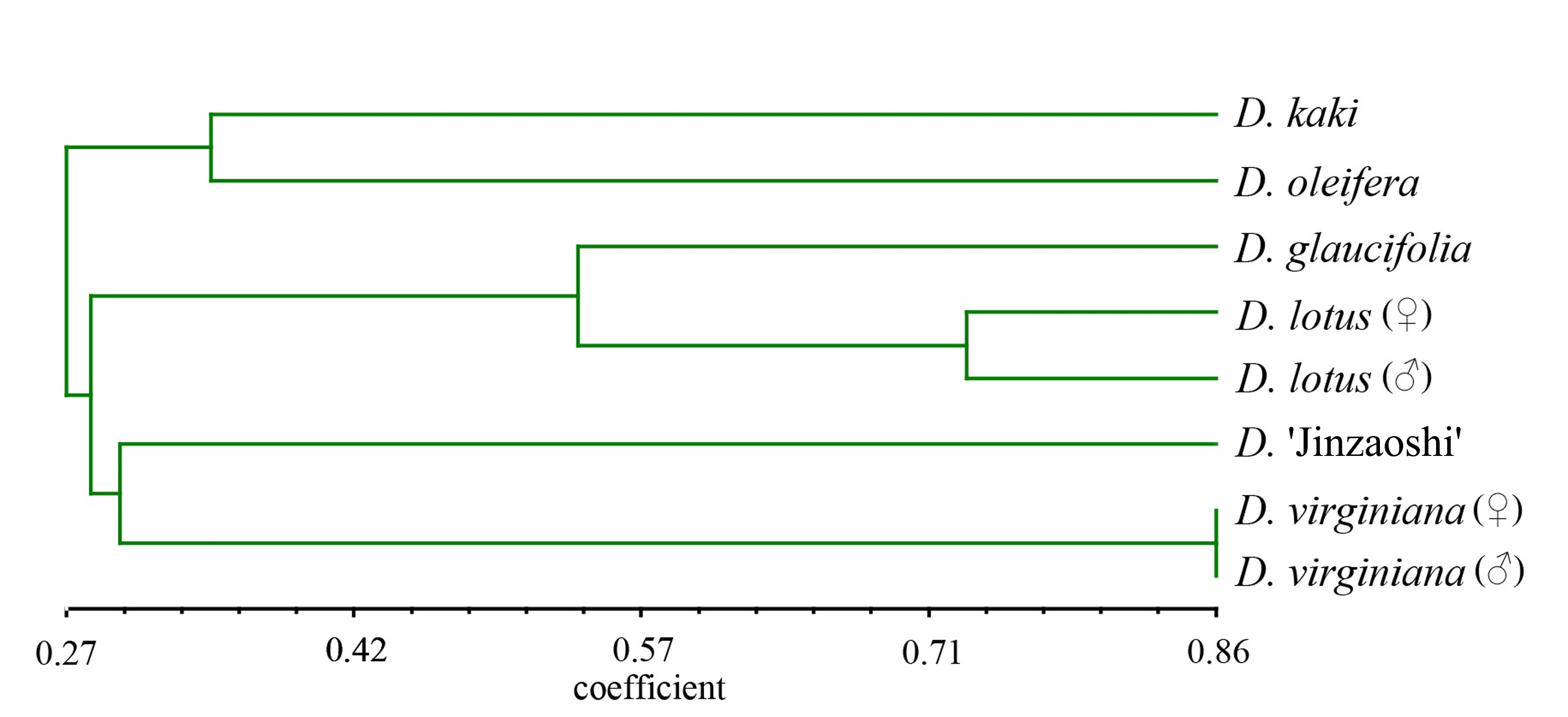

Supplement: S5 Fig — (TIF) [file pone.0159566.s011.tif]

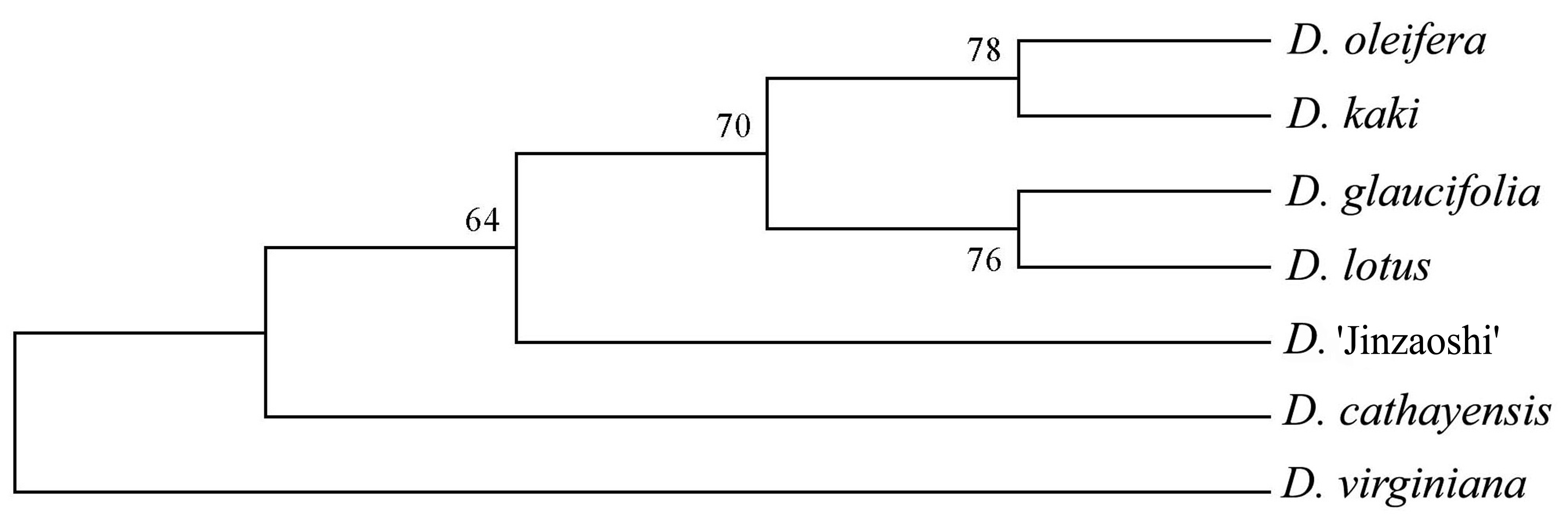

Supplement: S6 Fig — (TIF) [file pone.0159566.s012.tif]
